# Food storage facilitates professional religious specialization in hunter–gatherer societies

**DOI:** 10.1017/ehs.2022.17

**Published:** 2022-04-28

**Authors:** Joseph Watts, Elise M. Hamerslag, Cassie Sprules, John H. Shaver, Robin I. M. Dunbar

**Affiliations:** 1 Religion Programme, University of Otago, Dunedin 9016, New Zealand; 2 Center for Research on Evolution, Belief and Behaviour, University of Otago, Dunedin 9016, New Zealand; 3 Social and Evolutionary Neuroscience Research Group, Department of Experimental Psychology, University of Oxford, Oxford OX1 3UD, UK; 4 Department of Linguistic and Cultural Evolution, Max Planck Institute for the Science of Human History, Jena 07745, Germany

**Keywords:** Cultural evolution, hunter–gatherer, phylogenetic comparative methods, religious specialists, shaman, Social, anthropology

## Abstract

Professional religious specialists centralised religious authority in early human societies and represented some of the earliest instances of formalised social leadership. These individuals played a central role in the emergence of organised religion and transitions to more stratified human societies. Evolutionary theories highlight a range of environmental, economic and social factors that are potentially causally related to the emergence of professional religious specialists in human history. There remains little consensus over the relative importance of these factors and whether professional religious specialists were the outcome or driver of increased socio-cultural complexity. We built a global dataset of hunter–gatherer societies and developed a novel method of exploratory phylogenetic path analysis. This enabled us to systematically identify the factors associated with the emergence of professional religious specialists and infer the directionality of causal dependencies. We find that environmental predictability, environmental richness, pathogen load, social leadership and food storage systems are all correlated with professional religious specialists. However, only food storage is directly related to the emergence of professional religious specialists. Our findings are most consistent with the claim that the early stages of organised religion were the outcome rather than driver of increased socio-economic complexity.

## Graphical abstract

**Figure:**
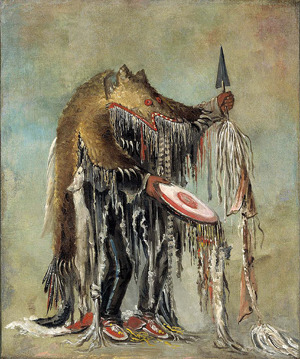

**Social media summary:** Food storage systems enabled professional religious specialists to emerge in hunter–gatherer societies

## Introduction

At the start of the Holocene, the world's religious landscape consisted of a diverse range of religious systems, each closely linked to local context (Bellah, 2011). Today, the majority of the world's population are adherents of a handful of globalised, doctrinal and organised religious traditions (Norenzayan, 2013). These changes represent a major transition in the scale and structure of religious, social and economic systems. Religious specialists facilitated the emergence of formal, systematised religious beliefs and behaviours, i.e. organised religion, by consolidating religious knowledge, standardising religious beliefs and coordinating ritual events (Rossano, 2007; Wright, 2010).

Religious specialists refer to individuals with a greater knowledge of, or ability to engage with, the supernatural than other community members. Broadly defined, religious specialists can include healers, mediums, shamans, priests and priestesses (Winkelman, 1986). These individuals are thought to represent one of the earliest kinds of formalised role differentiation in human societies (Rossano, 2007; Singh, 2017). Given the importance of religious specialists in the appearance of organised religion and social hierarchies, it is not surprising that scholars have suggested several hypotheses regarding their emergence and roles in human social evolution.

Existential insecurity theories claim that a central role of religious specialists is to explain and control existentially threatening and unpredictable events (Rossano, 2007; Singh, 2017). Consistent with these claims, religious specialists in non-industrial societies are called upon to heal disease and illness, and perform rituals to mitigate natural disasters (Winkelman, 1986). These explanations predict that religious specialists are more likely to arise in existentially threatening and unpredictable environments (Rossano, 2007; Singh, 2017), and are consistent with the claim that religions function to reduce stress, uncertainty and anxiety (Norris & Inglehart, 2011; Whitson & Galinsky, 2008).

A second family of theories suggest that the power and authority vested in religious specialists enabled them to establish social hierarchies and coordinate community behaviour (Fukuyama, 2011; Peoples et al., 2016; Rossano, 2007). Ethnographic and archaeological evidence has supported the claims that religious specialists organised ritual events (Fukuyama, 2011), reinforced group norms (Rossano, 2007) and facilitated cooperation (Peoples et al., 2016). These explanations predict that religious specialists preceded and facilitated socio-political complexity (Fukuyama, 2011; Peoples et al., 2016; Rossano, 2007), and are consistent with claims about the social functions of religion in human history (Norenzayan, 2013).

A third family of explanations argues that religious specialists are an outcome, rather than driver, of broad socio-cultural change (Testart et al., 1982; Winkelman, 1986). Religious specialists are suggested to emerge after shifts to more complex socio-economic environments, including greater social inequality and shifts in subsistence (Winkelman, 1986). These explanations predict that religious specialists are a product of increased socio-political complexity (Testart et al., 1982; Winkelman, 1986), and are consistent with the claim that religion is a reflection of socio-cultural conditions (Baumard & Boyer, 2013).

Existing cross-cultural studies differ over the precise forms of religious specialists they focus on, as well as how they define different forms of religious specialists. For example, some studies include healers, shamans and priests (Winkelman, 1986), while others focus specifically on shamans (Peoples et al., 2016; Singh, 2017). Of those studies focusing on shamans, some define the role of shamans as requiring specialists to enter a trance state (Singh, 2017), while others do not (Peoples et al., 2016). Despite differences in focus and definition, there remains substantial overlap in the explanatory targets of these studies, as well as a common interest in the professional status of religious specialists. Unlike non-professionals, professional religious specialists receive material payments for their services, which provide relief from regular subsistence activities and enable the development of more elaborate cultural practices (Smaldino, 2014). Material payments also indicate that the abilities and services of professional religious specialists are perceived as legitimate, necessary and valuable by their communities.

Methodological issues constrain existing inferences and conclusions about the evolution of professional religious specialists and their role in human cultural evolution. Support for each family of explanations often uses only specific cultural case studies (Atkinson, 1992; Fukuyama, 2011; Rossano, 2007). Cross-cultural comparative research has the potential to test the generality of explanations and the relative importance of causal factors across cultures (Mace & Pagel, 1994). Existing cross-cultural research on religious specialists across 47 societies suggests that religious specialists vary according to the society's primary means of subsistence and socio-political complexity (Winkelman, 1986; Winkelman & White, 1987), but the correlational methods used in these studies are subject to a number of methodological constraints. First, they do not identify the direction of causality between variables, so they cannot always distinguish between competing hypotheses. For example, a correlation between religious specialists and social inequality is consistent with the claim that religious specialists facilitate social inequality (Atkinson, 1992), as well as the claim that social inequality facilitates religious specialists (Winkelman, 1986). Second, they cannot model complex causal relationships between predictor variables. For example, these methods cannot reveal whether a relationship between environmental predictability and religious specialists is mediated by social leadership. Third, these methods assume that the units of analysis are independent, despite historical relationships between societies. This assumption has the potential to result in spurious correlations between traits (Mace & Pagel, 1994).

Phylogenetic comparative methods provide a family of statistical tools that can infer the evolutionary relationships between traits and statistically adjust for the common ancestry of societies (Mace & Pagel, 1994). When used appropriately, these methods can provide insight into the evolution of a diverse range of cultural phenomena, such as the cultural evolution of kinship (Jordan, 2011), language systems (Dunn et al., 2011), socio-political complexity (Currie et al., 2010), subsistence practices (Sheehan et al., 2018) and religion (Basava et al., 2021). Previous studies have used phylogenetic methods to reconstruct the history of shamanism in Tupi-speaking societies (Walker et al., 2012), to infer the evolutionary relationships between components of religion, including shamanism, in hunter–gatherer societies (Peoples et al., 2016), and test how features of religious and social systems have co-evolved in the history of the Pacific (Watts et al., 2015, 2016, 2018).

Here we use phylogenetic path analyses to test evolutionary theories of professional religious specialists in a global sample of hunter–gatherer societies. Confirmatory phylogenetic path analyses model directed dependencies between sets of variables (Hardenberg & Gonzalez-Voyer, 2013; van der Bijl, 2018), but can only compare a limited set of user-defined model structures. We extend these methods by identifying the best fitting models from the universe of possible model structures (see the Supplementary Materials). This approach to exploratory path analysis reduces researcher bias in model specification, makes minimal assumptions about path structures and can potentially identify novel and under-theorised causal pathways.

## Methods and results

We built the Hunter–Gatherer Religion Database (HGRD) (Watts et al., 2019) to test evolutionary theories of religion across a diverse sample of hunter–gatherer societies. The HGRD is a global database documenting the religious systems of 84 historic hunter–gatherer societies prior to widespread conversion to a major world religion, and prior to major shifts in subsistence. The HGRD is coded based on ethnographic source materials that describe the state of societies in the nineteenth and twentieth centuries. These relatively recent hunter–gatherer societies are not necessarily representative of deeper human history (Testart, 1988), but represent the kind of religious and social systems that can evolve within the constraints of hunter–gather subsistence. Here we combine variables on religion from the HGRD with established cross-cultural datasets from D-Place (Kirby et al., 2016).

**Figure 1.**
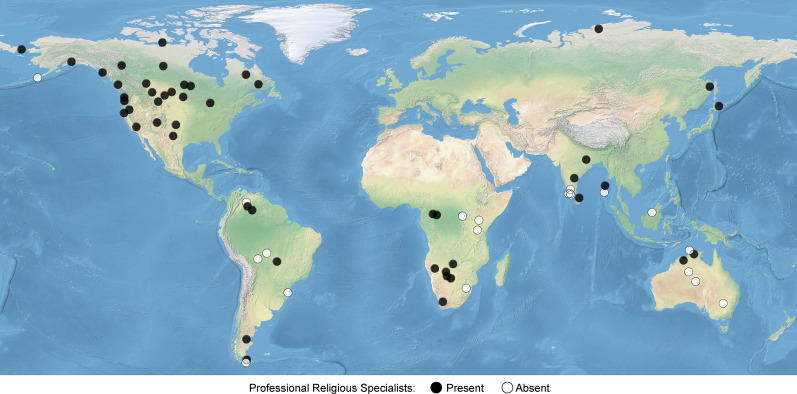
Geographic distribution of the 68 hunter–gatherer societies sampled in this study. There are 12 societies (18%) located in Africa, 6 (9%) in Australia, 14 (21%) in Eurasia, 26 (38%) in North America and 10 (15%) in South America.

In this study, we define professionals as individuals that serve a specialised role in the community and receive material compensation for their services, such as food, tools or currency. This definition can include shamans, witchdoctors, healers, magicians and sorcerers. Societies were coded based on published ethnographies. We found evidence of professional religious specialists in the majority of hunter–gatherer societies sampled. Of the 84 societies in the HGRD, professional religious specialists were found to be present in 48 societies and absent from 20 societies. Professional religious specialists were only coded as absent if ethnographic sources explicitly described their absence, or if there were substantial descriptions of role specialisation and religious systems that made their absence clear. There was insufficient information to code professional religious specialists in 16 societies and these societies were excluded from subsequent analyses. The remaining 68 societies were located across all inhabited world regions, other than Europe (Figure 1), and represent 35 different language families.

**Figure 2.**
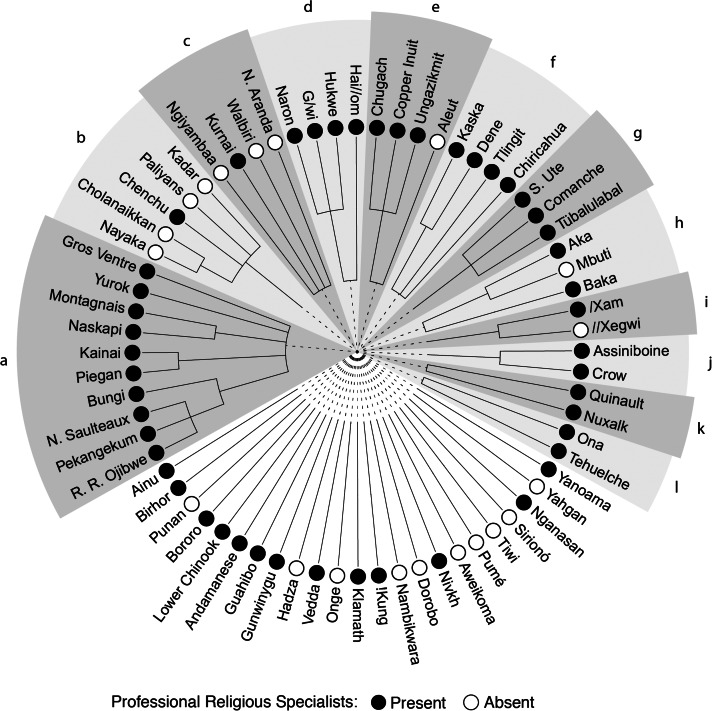
Distribution of hunter–gatherer societies with and without professional religious specialists, on a language-based phylogeny. Letters indicate language families; a, Algic; b, Dravidian; c, Pama–Nyungan; d, Khoe–Kwadi; e, Eskimo–Aleut; f, Athabaskan–Eyak–Tlingit; g, Uto–Aztecan; h, Atlantic–Congo; i, Tuu; j, Siouan; k, Salishan; and l, Chonan. Of the 10 North American societies speaking Algic languages (a), all 10 (100%) had professional religious specialists. In contrast, in the Dravidian language family of South Asia (b) only one in five (20%) societies had professional religious specialists.

Preliminary analyses found evidence of phylogenetic patterning of professional religious specialists on a language-based phylogenetic tree (*D* = −0.33, *p* = 0.001; Fritz & Purvis, 2010; Figure 2). The structure of this tree is based upon the classification of languages in the Glottolog (Hammarström et al., 2020) catalogue of languages (Supplementary Materials) and represents the branching of different languages over time. The distribution of professional religious specialists in our sample also shows evidence of geographic patterning (*z* = 446.75, *p* < 0.001). This suggests that both vertical and horizontal processes of transmission are potentially important forms of non-independence within our sample. We note that the phylogenetic and geographic distances between societies are correlated with one another (*z* = 112.15, *p* < 0.001), making it challenging to disentangle these two forms of non-independence. High rates of horizontal transmission can lead to inaccurate inferences when using phylogenetic methods to infer ancestral states of traits or the mode and tempo of trait change (Greenhill et al., 2009). The regression-based method of causal-path analysis used in this study does not require or assume that that a tree-like structure exists within the data (see Evans et al., 2021 for further discussion). We acknowledge that the distances represented on our language-based tree could potentially conflate vertical and horizontal relationships between societies, so we treat this tree as providing a general proxy for the non-independence of societies within our causal path analyses.

We conducted a search of contemporary literature on the evolution of religion and identified 15 hypotheses about the emergence of religious specialists (Table S1). To provide a preliminary test of these hypotheses, we use phylogenetic generalised least squares to identify the cultural and environmental factors associated with professional religious specialists (Table 1). These analyses are used to select variables for inclusion within our exploratory phylogenetic path analyses (Supplementary Materials).

**Table 1.** Phylogenetic generalised least squares results testing for the bivariate relationships between professional religious specialists and predictor variables. The phylogenetic correlation parameter estimate is represented by *α*.

Variable	Estimate	Standard error	*z-*Value	*n*	*p*	*α*
Social class	1.59	0.87	1.82	68	0.069	0.77
Slavery	0.98	0.9	1.09	68	0.276	0.7
Internal conflict	−0.09	0.55	−0.16	67	0.873	0.47
Intergroup conflict	0.37	0.58	0.64	68	0.523	0.7
Warfare	0.55	0.54	1.02	68	0.309	0.75
Food storage	2.59**	0.76	3.43	68	0.001	0.84
Social leadership	2.38**	0.73	3.25	68	0.001	0.87
Number of annual moves	−0.01	0.29	−0.03	55	0.98	0.75
Distance of annual moves	0.08	0.31	0.24	50	0.809	0.79
Consumer group size	0.49	0.34	1.46	65	0.144	0.8
Maximum camp group size	0.49	0.37	1.34	51	0.181	0.71
Total population size	0.36	0.27	1.34	68	0.179	0.71
Pathogen load	−1.08**	0.35	−3.12	68	0.002	0.91
Environmental richness	−0.57*	0.27	−2.1	68	0.036	0.46
Environmental predictability	0.92**	0.33	2.76	68	0.006	0.77
Environmental variation	0.02	0.26	0.06	68	0.952	0.65

*Indicates a *p-*value of less than 0.05, and ** indicates a *p-*value of less than 0.01.

The results of our bivariate phylogenetic generalised least squares analyses indicate that pathogen load (the prevalence of parasitic disease in an environment), environmental predictability (the predictability of the year-to-year seasonal changes in environmental carbon uptake) and environmental richness (the mean carbon uptake of an environment across all seasons) were significantly associated with professional religious specialists (Table 1). Contrary to existential insecurity theories, professional religious specialists were more likely to be found in environments with lower, rather than higher, pathogen load (Table 1 and H.14 of Table S1), and in more predictable environments (Table 1 and H.15 of Table S1). In contrast, professional religious specialists were more likely to be found in less rich environments (Table 1 and H.12 of Table S1). Together, these bivariate analyses provide mixed support for the claim that religious specialists function to explain, predict and control unpredictable life events (e.g. H.12–H.15 of Table S1), suggest a complex relationship between religious specialists and environmental factors and highlight the need for multivariate causal modelling.

Social leadership (the presence of widely recognised, general community leaders) and food storage (the presence of moderate, major or massive food stores) were also positively associated with professional religious specialists (Table 1). These bivariate analyses adjust for the non-independence of societies owing to common cultural ancestry, and are broadly consistent with a number of hypotheses (H.1, H.2, H.6., H.7 and H.8). However, these results do not account for potential co-variance between predictor variables, nor do they assess directionality. For example, the positive association between professional religious specialists and social leadership could be explained by the hypothesis that social leadership facilitates professional religious specialists (H.8) or the hypothesis that professional religious specialists facilitate social leadership (H.9). Analyses that can infer directional dependencies are therefore required.

We developed a novel method of exploratory phylogenetic path analysis to test for directional dependencies between professional religious specialists, and social, economic and environmental variables. This method builds on the frequentist model comparison functions implemented in the R package phylopath (van der Bijl, 2018), adding the ability to compare all possible model structures. This frequentist approach to model comparison is highly efficient and makes it computationally tractable to compare the fit of large numbers of path models. This is important because exploratory path analysis is computationally intensive and takes exponentially longer to perform for each additional variable included in the analyses. We performed exploratory phylogenetic path analysis with all variables found to have a significant bivariate relationship with professional religious specialists (Table 1). We then built an index of all possible models, with the restrictions that environmental predictability and environmental richness could not be predicted by other variables (other than each other), and that pathogen load could not be predicted by any socio-cultural variables. These restrictions improve the computational efficiency of analyses and avoid searching implausible path structures (see the Supplementary Materials for alternative analyses with fewer restrictions). Our exploratory path analyses searched through a total of 277,667 path models, and made no assumptions about the directionality of relationships between professional religious specialists and social and economic factors.

**Figure 3.**
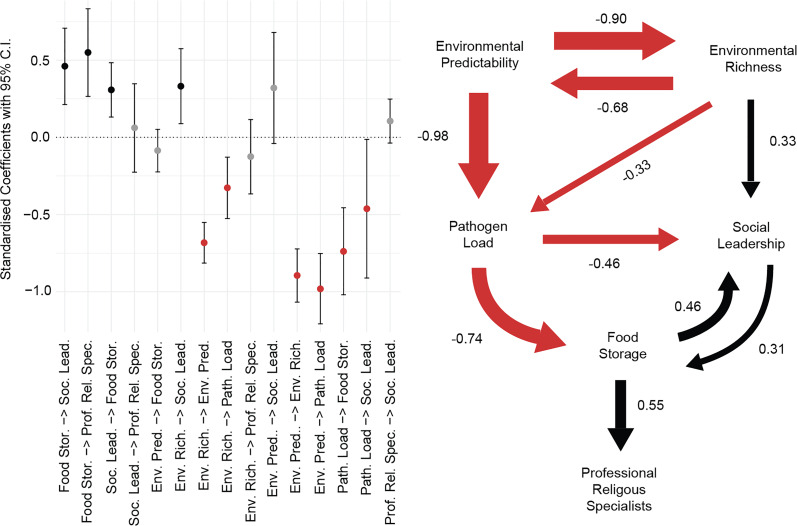
Summary model representing the average values across the best fitting path models. On the graph (left), points represent the standardised coefficients and bars represent their 95% CI. In the path diagram (right), arrow widths are proportional to path coefficients. Red arrows indicate negative coefficients, black arrows indicate positive coefficients and coefficients for which the 95% CI crosses 0 are not displayed.

We compared the fit of competing models using a modified version of the Akaike Information Criteria known as the *C* statistic Information Criterion corrected for small sample sizes (CICc) (Hardenberg & Gonzalez-Voyer, 2013). This metric is specifically designed for the comparison of path models owing to their typically high ratio of parameters to sample size. We summarise the conditional average of the best fitting models here (Figure 3), and full details of each of the best fitting models are available in the Supplementary Materials. In any specific path model, paths can only occur in one direction (e.g. social leadership → food storage), but our averaged model can include bidirectional paths in situations where the sample of best-fitting models includes arrows in both directions. Such cases (e.g. the relationship between social leadership and food storage in Figure 3) indicate that either/both directional dependencies are plausible. Of the 277,667 path models that we evaluated, there were 27 models within two CICc of the maximum (CICc = 48.49; Figure S1). After excluding paths with a 95% confidence interval (CI) that overlaps 0, the only variable that predicted the presence of professional religious specialists was food storage (Figure S2 and Table S5). Additional robustness checks are reported in the Supplementary Materials.

Food storage was the only variable robustly and directly related to religious specialists in our exploratory path analyses (Figure 3). Of the 34 hunter–gatherer societies in our sample coded as having food storage, 32 (94%) had professional religious specialists. In contrast, of the 34 hunter–gatherer societies without food storage, only 16 (47%) had professional religious specialists. In our best fitting models, food storage has a significant positive effect on professional religious specialists (Figure 3 and Figure S2). This is consistent with food storing societies being more likely to gain professional religious specialists. In societies that practised food storage, the types of food stored and the methods of preservation varied substantially across world regions. Siberian food storing societies such as the Samoyed primarily relied on salted and frozen stores of fish and reindeer (Hajdú et al., 1963), societies in California, such as the Yurok, generated stores of processed acorns (Kroeber, 1925), and South American societies, such as the Ona, stored dried meats and fungi (Cooper, 1946), although food storage tended to be less important in this region. Anthropologists and archaeologists have long speculated about the social and economic impacts of food storage on hunter–gatherer socio-economic systems (K. Flannery, 2002; Testart et al., 1982), and it has even been suggested that food storage played a bigger role in the Neolithic revolution than agriculture (Testart et al., 1982). These theories have not previously been rigorously tested using large-scale cross-cultural analyses, and food storage is largely absent from evolutionary explanations of religion.

For paid specialists to arise, societies must have both the economic and social conditions in place to allow for resource transactions. Food storage preserves food surpluses, and thus can increase the quantity of food available to exchange within a population. We note, however, that food was only one among many forms of payment that professional religious specialists received. Other forms of payment included weapons, rugs, clothing, homemade alcohol, blankets and horses (R. Flannery & Cooper, 1957, pp. 362–363; Howitt, 1887, p. 47; Kisliuk, 1998, pp. 110–112). This suggests that the effect of food storage on professional religious specialisation is not simply due to the availability of food as a form of currency.

Food storage many also facilitate resource exchange by breaking down collectivist social norms (Testart et al., 1982). Societies without food storage buffer against food shortages through reciprocal social networks of food exchange, both within and between bands. Food storage provides an alternative buffer against food scarcity, allowing communities to relax collectivist norms, and for the ownership, retention and exchange of resources to become more acceptable (Testart et al., 1982). In turn, these shifts in social norms enable paid social roles and the division of labour to emerge within communities (K. Flannery & Marcus, 2012).

Our sample included 16 hunter–gatherer societies with professional religious specialists but without food storage. Among some of these 16 societies, professional religious specialists appear to have received lower, less frequent and less reliable material payments. For example, Ainu shamans only received substantial material payments from wealthy community members (Ohnuki-Tierney, 1974). These cases suggest that material payments do not strictly entail that specialists received substantial relief from regular subsistence activities. Other societies with professional religious specialists, but not food storage, suggest alternative pathways to the emergence of professional specialisation. For example, the Vedda received payments from neighbouring agricultural and wage labourer-based societies (Seligman et al., 1911). While our causal model captures general trends in a sample of hunter–gatherer societies, there are few strict pathways in cultural evolution (Sheehan et al., 2018).

Environmental variables were significant components of all of our best fitting models, but were not directly related to professional religious specialists. The effect of pathogen load on professional religious specialists was found to be mediated by food storage, and the effects of environmental predictability and environmental richness were mediated by pathogen load and social leadership, which then led to food storage (Figure 3). The relationship between pathogen load and food storage suggests that societies with higher pathogen loads are less likely to develop food storage systems, perhaps owing to pathogen loads being greater in tropical climates where resource availability is more stable and food is more challenging to preserve.

The relationship between social leadership and professional religious specialists was also found to be fully mediated by food storage in our best fitting models (Figure 3). There was a positive bidirectional relationship between social leadership and food storage, suggesting that these two traits may have positive effects on one another. This relationship potentially reflects the need for social coordination in the construction and maintenance of food storage systems, and the ability of food storage to support role specialisation. In two of the 27 best fitting models, professional religious specialists were also found to have a positive effect on social leadership. This relationship is consistent with functionalist claims about the role of professional religious specialists in the socio-political evolution of small-scale societies (Rossano, 2007), but did not meet our criteria for inclusion in the average best fitting causal model.

These results challenge theories about the importance of existential insecurity in the evolution of religious systems. However, we also observe that the roles of religious specialists (both unpaid and paid) in hunter–gatherer societies often focus on explaining and controlling existentially threatening and unpredictable life events. For example, !Kung healers used trance dances to heal individuals and communities (Lee, 1967), Northern Saulteaux shamans were used to identify the number and position of hostile war parties (Skinner, 1912) and the Tübalulabal had rain doctors that specialised in performing rituals to control the weather (Voegelin, 1938). Counter-intuitively, our findings suggest that even though religious specialists generally dealt with unpredictable and threatening life events, more favourable environmental conditions facilitated the economic and social conditions that allowed a professional class of religious specialists to emerge.

## Conclusion

This study shows how comparative historical data and phylogenetic causal modelling can help untangle the complex pathways in human cultural evolution. Our findings are the most consistent with hypotheses in which the early steps towards the evolution of organised and hierarchical religions were primarily the result of, rather than driver of, changes in socio-economic complexity. Specifically, food storage may have been a catalyst of religious, economic and social change in hunter–gatherer societies. This possibility is corroborated by qualitative descriptions in ethnographies and previous theoretical literature, as well as the finding that food storage is the single strongest predictor of professional religious specialists within our bivariate analyses.

We note several ways in which future research could help develop and expand our research findings. First, we note that this study uses a broad classification of professional religious specialists that collapses a diverse range of religious roles. This helps identify common paths towards religious specialisation, and potentially means that these pathways generalise to other forms of specialisation. This breadth also comes at the costs of overlooking factors associated with specific forms of religious specialisation (Lightner et al., 2021). For example, different kinds of factors may be associated with the emergence of professional religious specialists that control weather, and those that perform healing rituals. Further research could test whether our findings apply to specific forms of professional religious specialisation. Second, we note that this study excluded 16 societies owing to a lack of ethnographic data on professional religious specialists. It is possible that societies without professional specialists were more likely to have been coded as missing data than societies with professional religious specialists. The reason for this is that ethnographers tend to focus on describing the features of societies that are present, not listing the features that are absent (Watts et al., 2021). This can potentially make it challenging to distinguish the absence of evidence from evidence of absence when coding ethnographic source materials. While we have no reason to believe that this would change the nature of our central findings, it potentially means that our dataset slightly over-represents the proportion of hunter–gatherer societies with professional religious specialists. This reflects a general challenge in the coding of cross-cultural comparative datasets that deserves further investigation. Third, we also note that while path analyses provide an effective way to model complex relationships between numerous variables, the direction of causal relationships inferred may not be as robust at those inferred through time series data and models. Future studies could build on our findings by modelling time-series data or using phylogenetic methods capable of inferring change over time (Evans et al., 2021).

These limitations notwithstanding, the method of exploratory path analyses developed here offers and additional tool for comparative research on both biological and cultural evolution. Our findings help clarify the complex pathway to the emergence of religious organisation and contribute to the growing recognition of the complexity of religious and social systems in hunter–gather societies (Boyd & Richerson, 2021).

## Research transparency and reproducibility

The full reproducible code and data are available on the Open Science Project page: https://osf.io/6jdqr/

## Supplementary material

For supplementary material accompanying this paper visit https://doi.org/10.1017/ehs.2022.17. click here to view supplementary material
