# Supplementary material for: Food storage facilitates professional religious specialization in hunter–gatherer societies
Source: Evol Hum Sci. 2022 Apr 28;4:e17. doi: 10.1017/ehs.2022.17 (PMC10426101; doi:10.1017/ehs.2022.17)
Supplement: Supplementary file 1 [file S2513843X22000172sup001.pdf]

Supplementary Materials

Food storage facilitates professional religious specialization in hunter-gatherer societies

Joseph Watts, Elise M. Hamerslag, Cassie Sprules, John H. Shaver and Robin I.M. Dunbar

Correspondence to: joseph.watts@otago.ac.nz

This PDF file includes:

Supplementary Methods

Supplementary Results

Figures S1 to S3

Tables S1 to S4

Our data, coding decisions, and the code used to process data and perform analyses are available through the projects Open Science Framework (OSF) page (https://osf.io/6jdqr/).

Supplementary Methods

Hypotheses

We undertook a literature search to identify hypotheses that attempt to explain the presence of religious specialists in historical small-scale societies. The literature we identified focuses on a wide range of different types of religious specialists and practitioners. For example, Singh (Singh, 2017) focuses on shamans, who are defined as "practitioners who enter trance to provide services" and are described to be a restricted professional class, and who are viewed as having greater abilities or powers to interact with invisible forces and diagnose or treat problems in life. By contrast, Winkleman (M. Winkelman, 1989) focuses on "magico-religious practitioners," who includes a broad range of healers, shamans and priests/priestesses that "had access to supernatural power". This means that the hypotheses we have identified in the literature may not always have been intended to apply specifically to professional religious specialists, as defined in this project. As such, we treat these hypotheses as a starting point for identifying the kinds of factors that could plausibly be causally related to the emergence of professional religious specialists in human history.

For previous research to contribute a hypothesis to our study, it needed to: (a) clearly identify one or more variables related to religious specialists in early human societies; (b) the proposed relationship(s) needed to plausibly apply across different world regions, and; (c) the variables identified needed to be operationalizable with available ethnographic source materials and databases. When multiple sources identified similar factors, and made similar predictions, this literature was collapsed into a single hypothesis. Table S1 provides a list of the 15 hypotheses we identified, along with selected quotes of text related to each hypothesis.

Variables

Below we outline the definitions used to code each variable used in this study. The full data and code used in this study is available through our OSF project page (https://osf.io/6jdqr/). The phylopath package assumes that variables are either continuous, or binary coded. For this reason, we dummy coded all categorical and ordinal variables. We also scaled numeric variables to have a standard deviation of one, and centered values to have a mean of zero.

Professional Religious Specialists

For an individual to be defined as a professional religious specialists they must: (a) be considered to have a special ability, expertise or authority in relation to the supernatural, and (b) receive material payments for their services. When members of communities are perceived to differ in supernatural abilities, expertise or authority, but do not receive material payments for their services, they were not considered professional religious specialists. For example, among the Pumé of South America, there was no "material gain to flow from attaining the position of shaman," so professional religious specialists were coded as absent (Petrullo, 1939). In cases where offerings were ostensibly made to gods or spirits, but clearly provided material benefits to religious specialists, these offerings were considered to be material payments to religious specialists. When there were both professional and non-professional religious specialists within a given society, professional religious specialists were coded as present. This variable was coded as part of our Hunter-gatherer Religion Database (Watts et al., 2019).

Social Class

Social class refers to the presence of social class distinctions within a society, including both wealth based distinctions and the stratification of the population into heritable social classes. Social class provides a general measure of the presence of social inequality within societies. This variable is a binary recoding of Binford's (Binford, Lewis, 2001) "CLASS" variable where 0 indicates social classes are absent, and 1 indicates that social classes are present. There are no missing data for this variable.

Slavery

Slavery refers to the state of slaves within a society, including debt slaves, other non-hereditary slaves, and hereditary slaves. This variable is a binary recoding of Binford's

(Binford, Lewis, 2001) “SLAVE” variable where 0 indicates that slavery absent and 1 indicates that slavery is present. There are no missing data for this variable.

Internal Conflict

Internal conflict refers to the frequency of internal conflict within a society. This refers to conflicts that occur within the largest consumer groups that regularly camp together within a year. This variable is a binary coding of Binford’s (Binford, Lewis, 2001) INTCON variable where 0 indicates that internal conflict is rare or absent and 1 indicates that internal conflict is moderate or frequent. There are two societies with missing data for this variable.

Intergroup Conflict

Intergroup conflict refers to the frequency of violence between groups that do not regularly or periodically camp together. Intergroup conflict includes raids conducted on groups of the same cultural unit that do no camp together. This variable is a binary coding of Binford’s (Binford, Lewis, 2001) GPGPCON variables where 0 indicates no violence reported, and 1 indicates that inter-group conflict was present. There is one society with missing data for this variable.

Warfare

Warfare refers to how widespread and frequent warfare is for a society. War can include periodic attacks on intruders as well as warfare undertaken to gain new territories. This variable is a binary recoding of Binford’s (Binford, Lewis, 2001) WAR1 variable where 0 indicates there is no organized warfare, and 1 indicates that organized warfare is present. There are no missing data for this variable.

Food Storage

Food storage refers to the quantity of food stored, including food use for special events and during low productive phases of the year. This variable is a dummy coding of Binford’s QTSTOR variable, which originally had four levels; 1 = minor quantities of food stored; 2 = moderate quantitates of food stored; 3 = major quantities of food stored; and 4= massive quantities of food stored. We binary coded this variable so that 0 indicates minor food storage, and 1 indicates moderate, major or massive food storage. There are no missing data for this variable.

Social Leadership

Social leadership refers to the presence of community leaders that are widely recognized, visible, and whose performance is evaluated by others. This does not include leadership based only on age-based distinctions, or domain specific differences in expertise. This variable is based on Binford's (Binford, Lewis, 2001) LEADER variable where 0 indicates that social leadership is absent, and 1 indicates that social leadership is present. There is no missing data for this variable.

Number of Annual Moves

Number of annual moves refers to the average number of times that household units shift locations per year. This variable is based on Binford's scalar NOMOV variable, which has value ranges from 0.1 to 45. For the purposes of our analyses we scale and center the NOMOV variable. There are 14 societies with missing data for this variable.

Distance of Annual Moves

Distance of annual moves refers to the average total distance that households move per year. This variable is based on Binford's DISMOV variable, which was originally in mile units and had values ranging from 8 to 570 miles (mean = 183.9, median = 90.0). The distribution of values for this variable was left skewed so we $\log_{10}$ transformed, scaled and normalized these values for the purposes of analyses. There are 19 societies with missing data for this variable.

Consumer Group Size

Consumer group size refers to the mean number of people that regularly camp together during the most aggregated season of the year. This variable is based on Binford's GROUP2 variable, which originally had values ranging from 20.2 to 330.0 (mean = 70.47, median = 46.50). The distribution of values for this variable was left skewed so we $\log_{10}$ transformed, scaled and normalized these values for the purposes of analyses. There are four societies with missing data for this variable.

Max Camp Size

Max camp size refers to the maximum number of people that camp together for subsistence purposes. This is a mean value and does not necessarily occur annually. This variable is based on Binford's GROUP3 scalar variable, which originally had values ranging from 62 to

1500 (mean = 230.77, median = 149.50). The distribution of values for this variable was left skewed so we $\log_{10}$ transformed, scaled and normalized these values for the purposes of analyses. There are 18 societies with missing data for this variable.

Total Population Size

Total population size refers to the total number of people within a cultural unit. These people do not necessarily interact with one another and this group size is based upon the classification boundaries of ethnographers. This variable is based on Binford's TLPOP scalar variable, which originally had values ranging from 70 to 13500 (mean = 1919.20, median = 1200.00). The distribution of values for this variable was left skewed so we $\log_{10}$ transformed, scaled and normalized these values for the purposes of analyses. There are no missing data for this variable.

Pathogen Load

Our variable on pathogen load is based on an established variables that Fincher and Thornhill (Fincher & Thornhill, 2008) generated from the Global Infectious Disease and Epidemiology Network (GIDEAN) (Yu & Edberg, 2005). This variable, known as "pathogen" in the "Binford" R package, refers to how common seven different classes of pathogens (totaling 22 different parasites) are within the region that the society is located. These seven classes of parasites were: leishmanias; trypanosomes; malaria; schistosomes; filariae; spirochetes; and leprosy. Each of these seven classes was coded on a three point scale by GIDEN or Fincher and Thornhill (Fincher & Thornhill, 2008), on which 3 means that the pathogen is endemic, 2 means it is sporadic, and 1 means that it is neither endemic nor sporadic. The sum of these seven categories was then taken as a measure of overall parasite prevalence. The data used to calculate these measures of pathogen load are based on observations gathered from April-June 2007, and have been shown to be highly correlated ($r = 0.77$) with measures of historical pathogen prevalence (Fincher et al., 2008). We centered and scaled pathogen scale scores for the purposes of analyses. There are no missing data for this variable.

Environmental Richness

Environmental richness refers to the mean grams of carbon uptake per square meter of land per month ($gC/m^2/month$). The rate of carbon uptake is a general measure of the ecological productivity of an environment, and is known as Net Primary Productivity (NPP). We use environmental data collected by NASA's Moderate Resolution Imaging Spectroradiometer

(Running et al., 1999), as processes and stored by the D-Place database of world cultures (Kirby et al., 2016). Further information about how mean NPP was calculated for each society is available on the D-Place website and in Kirby et al. (Kirby et al., 2016). There are no missing data for this variable.

Environmental Predictability

Environmental predictability refers to year-to-year variation in the cyclical patterns of NPP, as defined by Colwell's (Colwell, 1974) information theoretic index. An environment can be predictable if is highly stable (high constancy), or if there are highly regular seasonal changes (low contingency). For an environment to be unpredictable, it must have both low constancy and high contingency. We use D-Place's measure of NPP predictability (Kirby et al., 2016), which is calculated using data from NASA's Moderate Resolution Imaging Spectroradiometer (Running et al., 1999) and has been used in previous research on the cultural evolution of religion (Botero et al., 2014). Further information about how NPP predictability was calculated for each society is available on the D-Place website and in Kirby et al. (Kirby et al., 2016). There are no missing data for this variable.

Environmental Variation

Environmental variation refers to variation in NPP per month, as calculated and defined in the D-Place database (Kirby et al., 2016). Environmental variation is distinct from environmental predictability because environments can have large but predictable variation in NPP, such as those that occur over seasons, or large and irregular variation in NPP, such as intermittent droughts. The NPP values used here are calculated using data from NASA's Moderate Resolution Imaging Spectroradiometer (Running et al., 1999). There are no missing data for this variable.

Language Tree

We built a tree representing the historical relationships between the languages spoken in each society. This global tree was constructed using language classification data from the Glottolog catalogue of world languages (Hammarström et al., 2020). We assigned Glottocodes to societies in our sample using the codes assigned to Binford's sample in the D-Place database of world cultures (Kirby et al., 2016). Glottolog contains a comprehensive

index of classification data for over 20,000 world languages and dialects. Glottolog classifications are provided in a nested structure based on linguists' consensus judgements of the genealogical relationships between languages. This language classification provides the underlying structure of language phylogenies, but does not contain information on the scale of the branches. We developed a method for simplifying the classification scheme of Glottolog and scaling branches according to different potential distances between taxonomic classifications.

Our method of tree building restricts language classifications to four taxonomic levels; language family, language sub-family, language and dialect. Language family refers to the broadest taxonomic grouping within Glottolog. Language isolates are each treated as being of their own distinct language family. There are a total of 37 language families included in our sample, including the Austronesian, Dravidian and Uto-Aztcan language families. We define a language sub-family as the highest order taxonomic classification within a language family that differentiate at least 10% of the documented languages within that language family. We follow Glottolog classification codes for languages and dialects. Two societies in our sample, Cholanaikkan and Nayaka, are classified as speaking the Jennu Kurumba language. For the purpose of representation on our language phylogeny, we treat these two societies as speaking different dialects of the same language.

We built three trees, each representing a different approach to scaling the distances between societies within our phylogenetically adjusted models (Figure S3). These three trees provide a proxy for cultural ancestry within phylogenetic based statistical methods and we replicate analyses across all three to test the sensitivity of our results to branch scaling assumptions. We emphasize that the scaling of branches on these three trees is not proportional to the timing of language diversifications in human history. Across all trees, the root of a tree is a polytomy with each language family diverging. All language isolates are treated as representing different language families. In the first tree, the phylogenetic distance between languages increases linearly with taxonomic distance. The length of branches representing the language family splits from the root of the tree have a length of one, branches representing the splitting of language subfamilies from families have a length of one, and branches representing the splitting of languages from subfamilies have a length of one. Branches representing the distance between dialects and languages was set to 0.5, and the branch representing the length from the parent language to the language sub-family was scaled to 0.5 in order to retain ultrametricity. In this tree, two dialects of the same language

have a distance of one, two languages of the same language sub-family have a distance of two, two languages from different sub-families of the same language family have a distance of four, and two languages from different language families have a distance of six.

In the second tree (Tree 2 in Figure S3), the phylogenetic distance between languages progressively increases according to the taxonomic distance between languages. The length of branches representing the language family splits from the root of the tree have a length of three, branches representing the splitting of language subfamilies from families have a length of two, and branched representing the splitting of languages from subfamilies have a length of one. Branches representing the distance between dialects and languages was set to 0.5, and the branch representing the length from the parent language to the language sub-family was scaled to 0.5 in order to retain ultrametricity. In this tree, two dialects of the same language have a distance of one, two languages of the same language sub-family have a distance of two, two languages from different sub-families of the same language family have a distance of six, and two languages from different language families have a distance of twelve.

In the third tree (Tree 3 in Figure S3), the phylogenetic distance between languages increases sub-linearly with the taxonomic distance between languages. The length of branches representing the language family splits from the root of the tree have a length of one, branches representing the splitting of language subfamilies from families have a length of two, and branched representing the splitting of languages from subfamilies have a length of three. Branches representing the distance between dialects and languages was set to 1.5, and the branch representing the length from the parent language to the language sub-family was scaled to 1.5 in order to retain ultrametricity. In this tree, two dialects of the same language have a distance of three, two languages of the same language sub-family have a distance of six, two languages from different sub-families of the same language family have a distance of ten and two languages from different language families have a distance of twelve.

This tree building approach results in a series of three simplified, ultrametric phylogenies that provide a proxy for the ancestral relationship between languages. In the main manuscript, we report only the results using the first tree, discuss the results of our replicated analyses in the Supplementary Results section, and provide the outputs on the OSF project page (https://osf.io/6jdqr/). Our findings remain largely unchanged across these three trees, supporting the generality of our findings.

Exploratory phylogenetic path analysis

Standard path analyses are unable to account for the non-independence of societies, so are subject to Galton's Problem (Mace & Pagel, 1994). Phylogenetic comparative research methods have the ability to help address Galton's Problem by incorporating a family tree representing the historical relationships between cultures (Mace & Pagel, 1994). The method of confirmatory path analysis developed by Hardenberg and Gonzalez-Voyer (Hardenberg & Gonzalez-Voyer, 2013), and implemented in the R package phylopath (van der Bijl, 2018), provides a framework for comparing different causal path structures while adjusting for the phylogenetic non-independence of societies. This method provides a way to model and compare a defined set of path models representing the causal relationships while addressing Galton's Problem.

We build on existing the confirmatory phylogenetic path analysis method by developing a package called BEPA (Brute-force Exploratory Path Analysis). The BEPA package includes functions to builds an index of all possible causal path structures for a set of variables, and to convert models from formulae to directed acyclic graphs. The approach taken to indexing all possible causal paths is computationally intensive and the number of possible models increases exponentially with each additional variable included. In order to improve performance, this package includes an option argument to exclude models that contain certain kinds of path relationships. For example, users can choose to generate an index of all possible models, except those in which Social Leadership predicts environmental predictability. This, and the multi-threading argument, aid in efficiency but we warn users that the package remains computationally intensive. The code used to perform BEPA is publicly available through GitHub (https://github.com/Joseph-Watts/BEPA).

In the main article we summarize the best fitting models from our analyses. The best fitting models are defined as those models within 2 CICc of the best fitting model (36, 43). Averaging across the best fitting models accounts for uncertainty in model selection and conditional averaging calculates average path coefficients only when they are included in a model (rather than treating absent paths as zero) (36, 43).

Supplementary Results

Phylogenetic signal

We tested for phylogenetic patterning of professional religious specialists on our language phylogeny by calculating the Fritz & Purvis (Fritz & Purvis, 2010) D statistic using the phylo.d function from the R package named caper (Orme et al., 2013). The number of permutations was set to 100,000.

Phylogenetic and geographic distances

We examined regional clustering in professional religious specialists and generated a matrix representing the geographic distances between societies. The geographic distribution of professional religious specialists suggests differences in the frequency of professional religious specialists across world regions (Figure 2). Of the 26 hunter-gatherer societies located in North America, 25 (96%) were found to have professional religious specialists, whereas 2 of the 6 (33%) hunter-gatherer societies in Australia had professional religious specialists.

Using a Mantel test, we found evidence of significant geographic clustering in the distribution of professional religious specialists (z = 446.75, p = 0.001). Mantel tests (z = 19.00, p = 0.001) and Fritz and Purvis D (D = -0.33, p = 0.001) also indicate that there is phylogenetic signal in the distribution of professional religious specialists. These tests suggest that there is geographic and spatial autocorrelation in the distribution of professional religious specialists that must be adjusted for in comparative analyses (Dow & Eff, 2008; Jordan, 2013). We note that the geographic and phylogenetic distances between societies are themselves significantly correlated (z = 112.15, $p < 0.001$). Here we use phylogenetic comparative methods to adjust for the non-independence of societies, treating phylogenetic distance between societies as a general proxy for the non-independence of societies in our sample.

Non-Phylogenetic Models

In addition to the phylogenetically adjusted models reported in the main article, we performed a series of standard Generalised Linear Models (GLMs). These models tested the bivariate relationship between predictor variables and our variable professional religious specialists, without adjusting for the common ancestry of societies using a phylogeny. GLM analyses were performed using the GLM function in R (R Core Team, 2015), with a binomial distribution.

Of the 16 variables analyzed using GLMs, seven were found to be significantly correlated with Professional Religious Specialists in hunter-gatherer societies (Table S2). By contrast, only five of these relationships remained statistically significant after adjusting for common ancestry within our phylogenetically adjusted GLMs (Table 1). The results of our GLMs are included to show the importance of adjusting for common ancestry when modelling the cross-cultural distribution of professional religious specialists. We do not discuss the results of the GLMs further because this statistical method assumes an independent sample, which the results of our phylogenetic analysis do not support (see the section below on Tree Construction).

Phylogenetically-adjusted Bivariate Relationships

As reported in our main analyses, Phylogenetic Generalized Linear Models (PGLMs) were used to identify the cultural and environmental factors associated with professional religious specialists (Table 1). Phylogenetically adjusted bivariate analyses were performed using the phyloglm function in the phylolm R package (Ho et al., 2016). For our PGLM analyses we used logistic MLPE as the method of estimation and set the tolerance of the fitted values to 30. These analyses do not account for potential co-variance between predictor variables, but do adjust for dependence due to common cultural ancestry. In order to understand how adjusting for the non-independence of societies affects our results, we also include standard non-phylogenetic bivariate analyses in our Supplementary Methods (Table S2).

The results of our bivariate PGLM analyses are reported in the main article. One limitation of these bivariate analyses is that they do not assess the directionality of the relationship between variables, so cannot always distinguish between competing hypotheses. For example, the positive association between social leadership and professional religious specialists is compatible with the hypothesis that professional religious specialists facilitate

social leadership (H.7), as well as the hypothesis that social leadership facilitates professional religious specialists (H.8). Another limitation of these analyses is that it is not possible to tell whether the effects of one variable on another is partially of fully mediated by another. To address these limitations, we performed phylogenetically adjusted path analyses (see the main article and the exploratory phylogenetic path analysis section of the Supplementary Methods).

Follow-up analyses

We performed a series of follow-up analyses to test how robust the results of our exploratory phylogenetic path analyses (main article) are to variations in tree structure and model constraints.

First, we replicated our analyses using the second and third trees described in the Language Tree section of the Supplementary Methods (Tree 2 and Tree 3 in the Figure S3). In our first replication, using Tree 2 (Figure S3), the results of our phylogenetically adjusted bivariate analyses find the same variables are significantly associated with professional religious specialists, and the direction of their relationship remains the same. For the exploratory phylogenetic path analyses using Tree 2, there are 19 best fitting models (within 2 CICc of the lowest CICc). Across all 19 of these best fitting causal models, the only variable that is significantly and directly causally linked to professional religious specialists is food storage. In all 19 best fitting models the direction of this relationship is the same as that found using Tree 1.

The result of our second replication, using Tree 3 (Figure S3), were also highly consistent with the results obtained using Tree 1 and Tree 2. In our bivariate phylogenetic analyses, we find that that the same variables are significantly associated with professional religious specialists, and in the same direction. For the exploratory phylogenetic path analyses using Tree 3, there are 15 best fitting models (within 2 CICc of the lowest CICc). As is the case with Tree 1 and Tree 2, across all of these best fitting causal models, the only variable that is significantly and directly causally linked to professional religious specialists is food storage. In all 15 of the best fitting models the direction of this relationship is also the same as that found using Tree 1 and Tree 2.

We also replicated our exploratory phylogenetic path analyses using Tree 1 without any restrictions on the possible causal paths. This means that cultural variables, such as professional religious specialists, could be predictors of environmental variables, such as environmental predictability. This unrestricted analysis was highly computationally intensive and involved testing the fit of a total of 3,771,782 possible model structures. This includes a large number of theoretically implausible models, such as models in which professional religious specialists affect environmental predictability. The results of these models found 24 models within 2 CICc of the best fitting model. Of these best fitting causal models, the only variable that is significantly and directly causally linked to professional religious specialists is food storage. In 22 of the 24 best fitting models the direction of the causal path was the same as that reported in the main text (Food Storage → Professional Religious Specialists). However, in two of the best fitting models the direction of the causal path was reversed (Professional Religious Specialists → Food Storage). These two models were the 16th (m1852899) and 17th (m1852829) best fitting models and were 1.67 and 1.77 CICc away from the best fitting model. This means that while these models meet the threshold for inclusion in the best fitting models, they represent only a small proportion of the total sample of best fitting models and are assigned relatively low weighting.

The results of our follow up analyses indicate that the central findings of our study hold when using alternative approaches to branch length scaling, and without placing restrictions on the path structures of models. Full details of these follow up analyses, including reproducible code, are available through the OSF project page (https://osf.io/6jdqr/).

Supplementary Figures

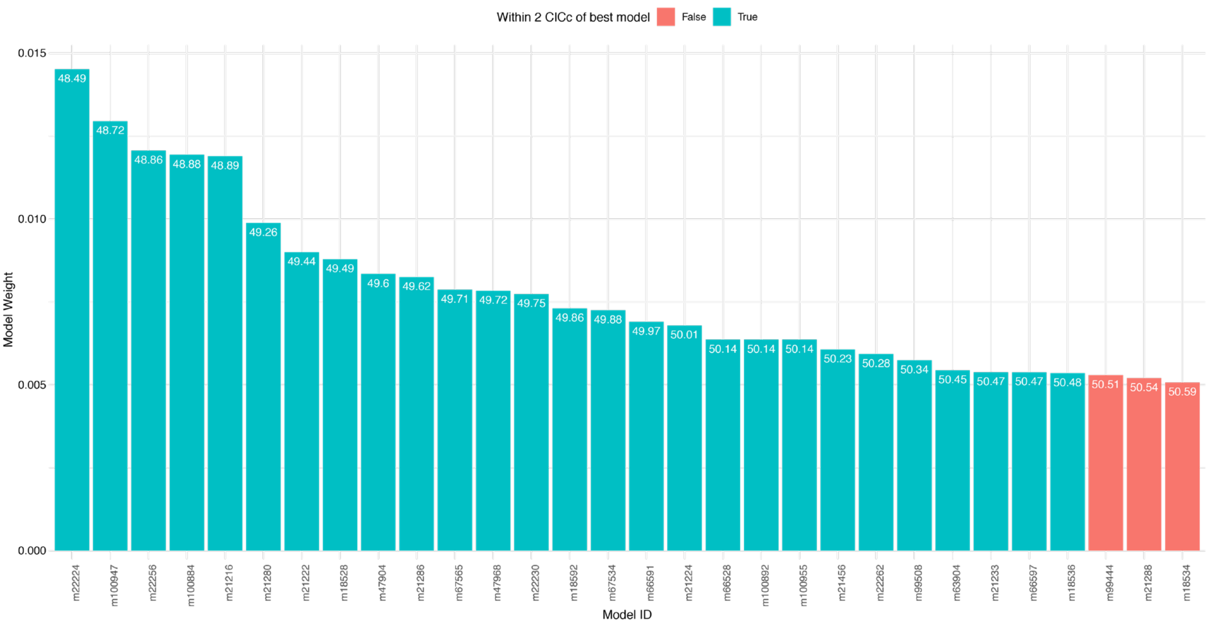

Figure S1. Model weights of the 30 best fitting causal path models. Values at the top of bars represent the model CICc.

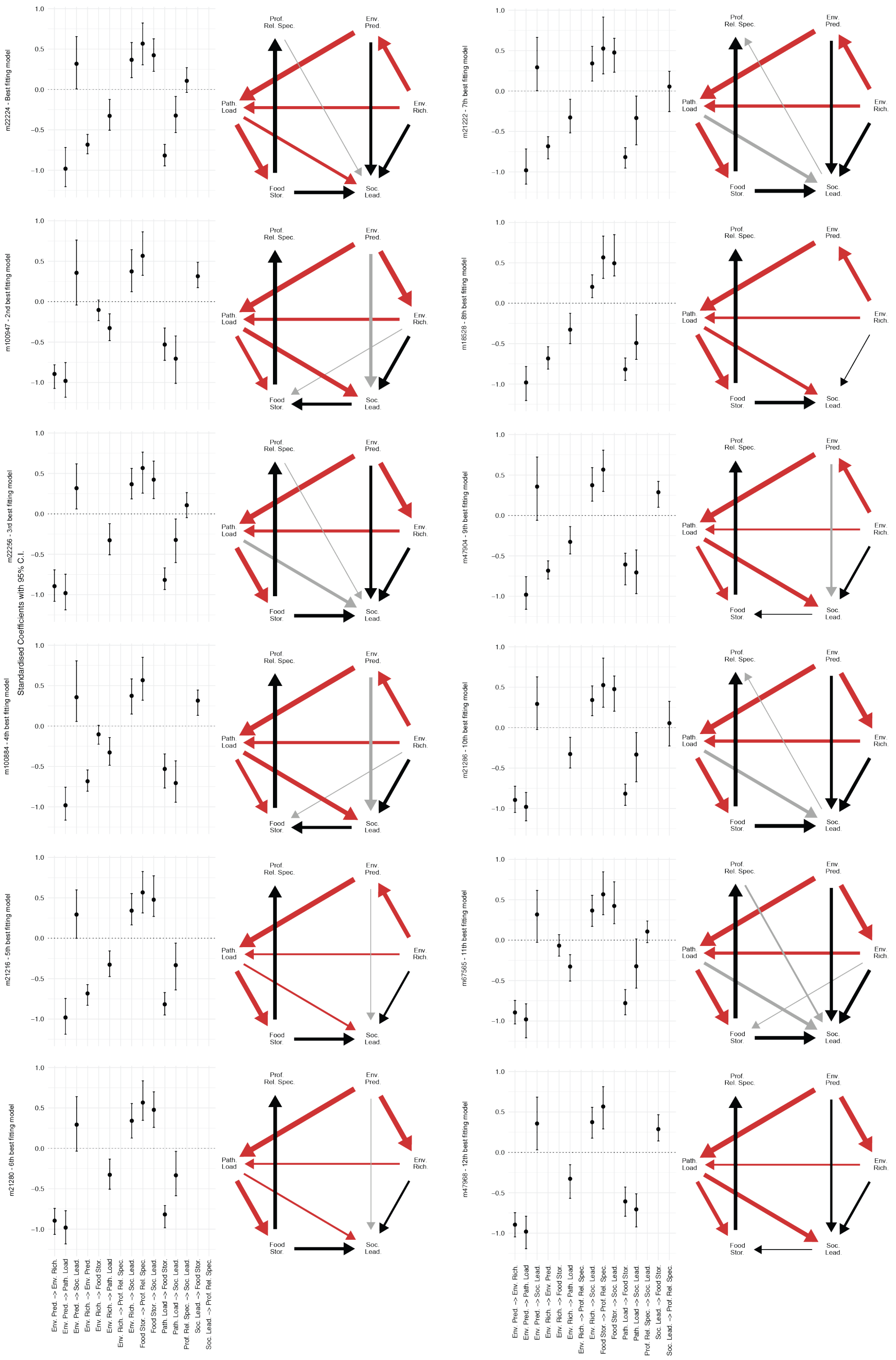

Figure S2 Part A.

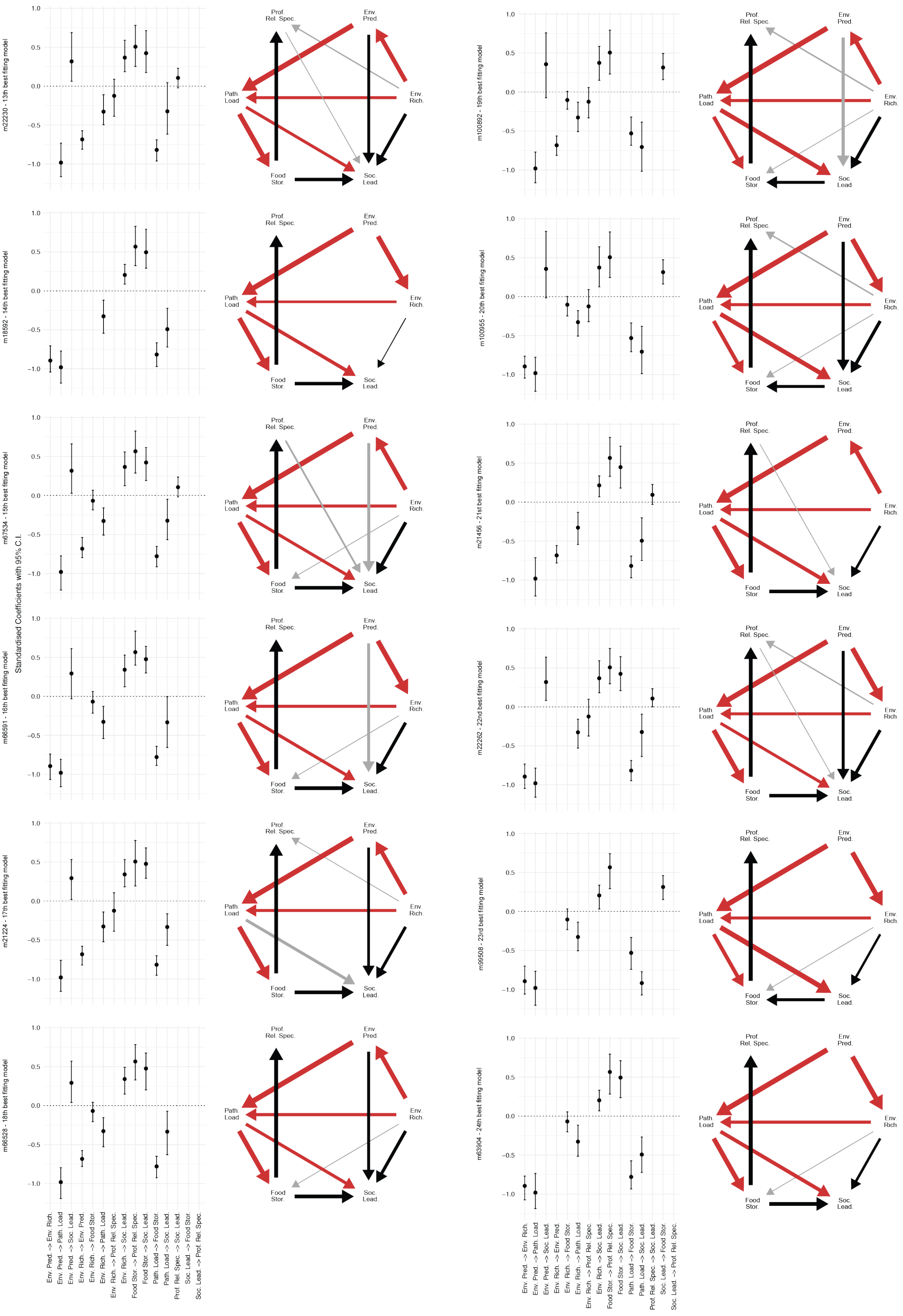

Figure S2 Part B.

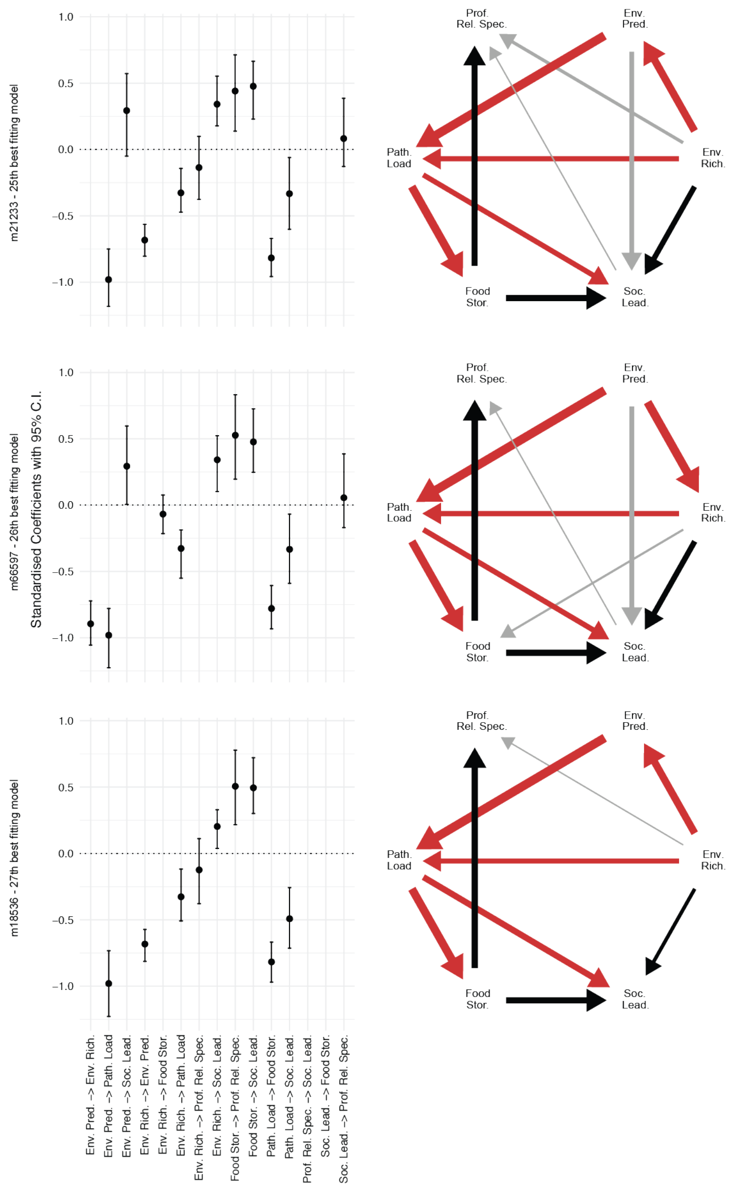

Figure S2 Part C. The 27 models within 2 CICc of the best fitting model. On the graphs, points represent the standardized coefficients and bars represent their 95% CI. In the path diagrams, arrow widths are proportional to path coefficients. Red arrows indicate negative coefficients, black indicate positive coefficients, and grey indicate coefficients for which the 95% CI crosses 0.

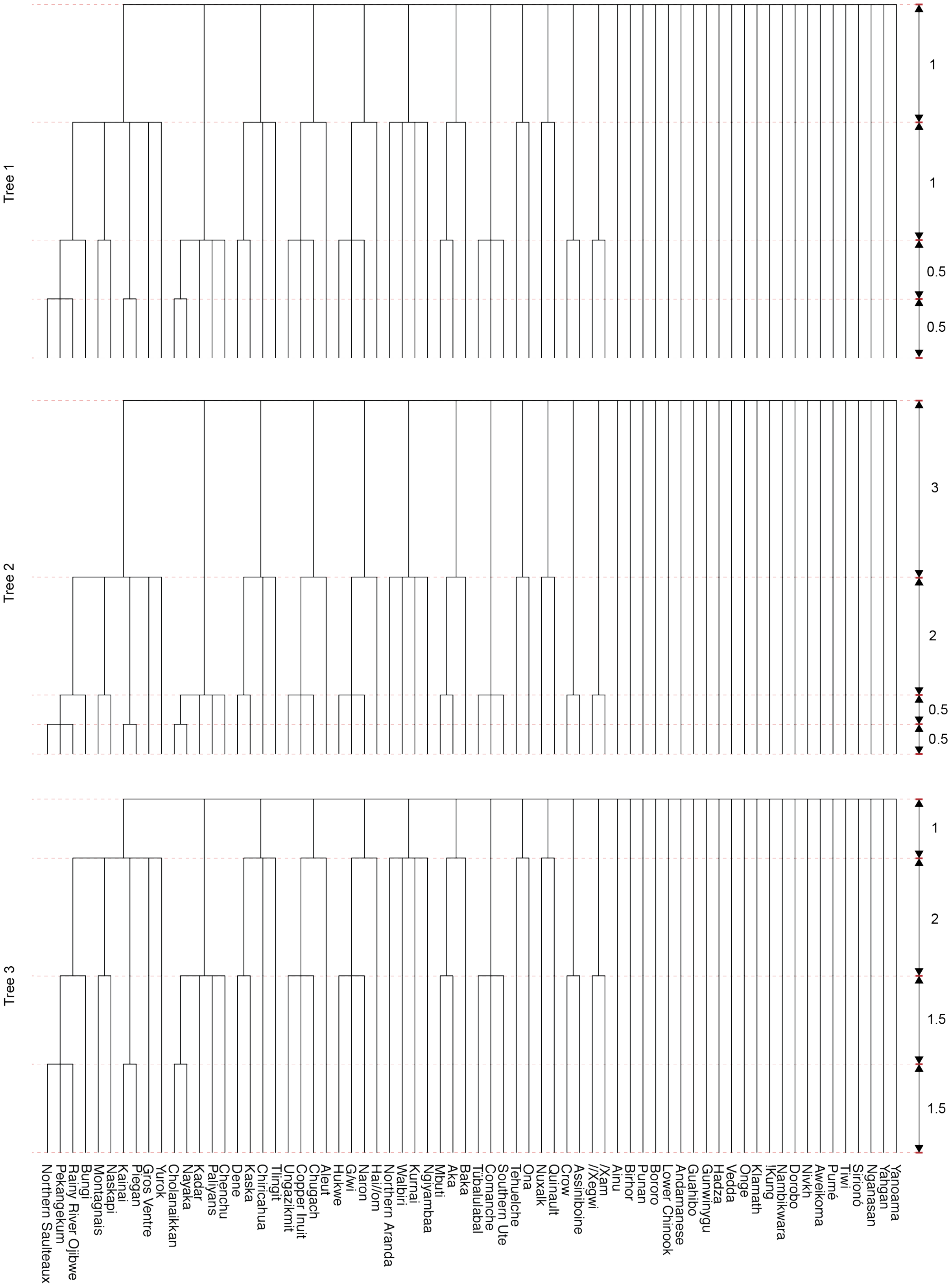

Figure S3. Branch length scaling of the three different trees used in this study. The results reported in the main text are from Tree 1 and the results of Tree 2 and 3 are provided in the Supplementary Results.

Supplementary Tables

Table S1. Index of 15 hypotheses about the cultural and ecological factors associated with professional religious specialists in small-scale human societies. Relationships indicate the direction of the predicted causal relationship between religious specialists and each other variable.

H.	Relationship	H. Source	Variable(s) Used
**1**	RS → Social Inequality (positive effect)	"Soviet scholars regard shamanism as a form of religious practice in pre-class societies. Some posit an early collective stage in which shamanic practice was open to anyone, followed by the development of professional specialization. As specialists who demanded payments and sacrifices from clients, shamans served to promote and legitimate growing economic inequality and the rise of feudal society." (Atkinson, 1992)	Social Classes
		"What we suggest, then, is that this emphasis on spiritual authority and power is one of several strategies for acquiring power that has a number of advantages for aggrandizers in transegalitarian and chiefdom communities …. by using spiritual power as a subterfuge to justify and control excess resources and labor, to blunt possible accusations of self-aggrandizement, and as a commodity that cannot be stolen, aggrandizers could circumvent a number of problems in trying to promote their self-interests." (Hayden & Villeneuve, 2010)	Slavery

**2**	Social Inequality → RS (positive effect)	"the central forces transforming shamanism are a sedentary-agriculture lifestyle, the process of political integration of local communities into politically stratified societies, and the presence of classes (with castes or hereditary slavery)" (M. J. Winkelman, 1990)	Social Classes
		"The initial hypothesis that systematic differences in magico-religious practitioners occur as a function of social complexity is refined to suggest that agriculture, political integration, and the presence of social classes are the specific conditions that are responsible for the creation of the different types and configurations of magico-religious practitioners….. A second major aspect of the magico-religious phenomenon is the control of social power in sedentary agricultural societies, particularly those with hierarchical political integration." (M. Winkelman, 1986)<br><br>"The notion that religion has its origin in society or social expression is consistent with the findings here, with the Priest role arising as a consequence of social and economic conditions (agriculture, political integration). However, the activities of the Shaman and related trance practitioners cannot be seen as arising out of religion in any fundamental sense … although these experiences are apparently shaped by social conditions (e.g., soul flight versus possession; see Bourguignon and Evascu 1977; Winkelman 1984, chapter 6;1986), the basic altered state that gives rise to the role and capacity is not determined by culture or social experience, but by human psychophysiology." (M. Winkelman, 1986)	Slavery

		"Becoming a shaman also provided a way for low-status individuals to attain prestige, such as in some hierarchical societies of the Pacific Northwest (Gunn 1966), while in other instances, shamans were regarded as attractive sexual partners." (Singh, 2017)	
**3**	RS → Cooperation (positive effect)	"Shamans are healers, ritual leaders, and influential members of society whose keen insight and success in solving social problems (Rossano 2007; Winkelman 1990) can lead to wealth, power, and access to mates. Shamanism acts as a mechanism to reinforce social norms, encouraging group cooperation through ritual and social bonding, and calming anxiety during times of resource stress (Hayden 1987; Rossano 2007; Winkelman 1990; Winkelman 2010)." (Peoples et al., 2016)<br><br>"By binding supernatural authority to the punishments incurred when taboos are broken, the shaman reinforces group norms. Whatever the specific task, the good shaman serves as an emissary to the supernatural world, intent on repairing or enhancing the community's relationship to the supernatural and to each other—or, as Vitebsky (2000, pp 63) puts it, 'The mystic is also a social worker." (Rossano, 2007)	Internal Conflict
**4**	Outgroup warfare → RS (positive effect)	"According to this theory, and the writings of many other researchers (Malinowski 1948), magical practices result from individuals trying to control important, uncontrollable events. As	Intergroup Conflict<br>Warfare

		individuals are better able to deal with this uncertainty – or if they confront less of it – they will depend less on shamans. Although a world completely devoid of uncertain outcomes seems implausible, Hart and Pilling (1960) offered this hypothesis to explain the absence of shamanism among the Tiwi. Citing the adequate rainfall, the consistent food supply, a dearth of dangerous animals, the rarity of tropical diseases, climatic docility, and the absence of antagonistic neighbors, the ethnographers concluded, "[The Tiwi] never invented magic to control their environment because their physical environment was on the whole a satisfactory and not a hostile universe" (p. 88).” (Singh, 2017)	
**5**	RS → Outgroup warfare (positive effect)	"Religious authority and military prowess go hand in hand. Religious authority allows a particular tribal leader to solve the large-scale collective action problem of uniting a group of autonomous tribes. To a much larger degree than economic benefit, religious authority can explain why a free tribal people would be willing to make a permanent delegation of authority to a single individual and that individual’s kin group. The leader can then use that authority to create a centralized military machine that can conquer recalcitrant tribes as well as ensure domestic peace and security, which then reinforces the leader’s religious authority in a positive-feedback loop." (Fukuyama, 2011)	Intergroup Conflict<br>Warfare
**6**	Food storage → RS (positive effect)	“The Neolithic Revolution is believed to have paved the way for civilization, class society, and the state. The basic assumption is	Food Storage

		always that only agriculture was able to generate a regular economic surplus sufficient to maintain a nonproductive class, such as priests, warriors, bureaucrats, and the like….This view, however, can no longer be maintained, for quantitative studies (e.g. Lee 1968, 1969) show that, contrary to what has generally been assumed, hunter-gatherers do not work hard to make a living…. The explanation in terms of surplus definitely has to be replaced by a new one… preliminary evidence suggests that the relevant factor for the development of inequalities is not the presence or absence of agriculture, but the presence or absence of a storing economy, whether it be hunting-gathering or agricultural.” (Testart et al., 1982)	
**7**	Sociopolitical leadership → RS<br>(positive effect)	“the central forces transforming shamanism are a sedentary-agriculture lifestyle, the process of political integration of local communities into politically stratified societies, and the presence of classes (with castes or hereditary slavery)” (M. J. Winkelman, 1990)<br><br>“The initial hypothesis that systematic differences in magico-religious practitioners occur as a function of social complexity is refined to suggest that agriculture, political integration, and the presence of social classes are the specific conditions that are responsible for the creation of the different types and configurations of magico-religious practitioners….. A second major aspect of the magico-religious phenomenon is the control of social power in sedentary agricultural societies, particularly those with hierarchical political integration.” (M. Winkelman, 1986)	Social Leadership

		“The notion that religion has its origin in society or social expression is consistent with the findings here, with the Priest role arising as a consequence of social and economic conditions (agriculture, political integration). However, the activities of the Shaman and related trance practitioners cannot be seen as arising out of religion in any fundamental sense … although these experiences are apparently shaped by social conditions (e.g., soul flight versus possession; see Bourguignon and Evascu 1977; Winkelman 1984, chapter 6;1986), the basic altered state that gives rise to the role and capacity is not determined by culture or social experience, but by human psychophysiology.” (M. Winkelman, 1986)	
**8**	RS → Sociopolitical leadership<br>(positive effect)	"certain quality of an individual personality, by virtue of which he is set apart from ordinary men and treated as endowed with supernatural, superhuman, or at least specifically exceptional powers or qualities. These are such as are not accessible to the ordinary person, but are regarded as of divine origin or as exemplary, and on the basis of them the individual concerned is treated as a leader" (Weber, 1958)<br><br>“There is evidence that in shamanism lie the origins of formal politics.... though there have been societies with shamans with no acknowledged political leaders, there have been few if any societies with a political leaders but no religious experts. And in some	Social Leadership

		societies the shaman and the political leader have been one and the same.” (Wright, 2010)	
**9**	Sedentism → RS (positive effect)	“the central forces transforming shamanism are a sedentary-agriculture lifestyle, the process of political integration of local communities into politically stratified societies, and the presence of classes (with castes or hereditary slavery)” (M. J. Winkelman, 1990)	Number of Annual Moves
		“The initial hypothesis that systematic differences in magico-religious practitioners occur as a function of social complexity is refined to suggest that agriculture, political integration, and the presence of social classes are the specific conditions that are responsible for the creation of the different types and configurations of magico-religious practitioners….. A second major aspect of the magico-religious phenomenon is the control of social power in sedentary agricultural societies, particularly those with hierarchical political integration.” (M. Winkelman, 1986)	Distance of Annual Moves
		“The notion that religion has its origin in society or social expression is consistent with the findings here, with the Priest role arising as a consequence of social and economic conditions (agriculture, political integration). However, the activities of the Shaman and related trance practitioners cannot be seen as arising out of religion in any fundamental sense … although these experiences are apparently shaped by social conditions (e.g., soul flight versus possession; see Bourguignon and Evascu 1977;	

		Winkelman 1984, chapter 6;1986), the basic altered state that gives rise to the role and capacity is not determined by culture or social experience, but by human psychophysiology.” (M. Winkelman, 1986)	
**10**	Population size → RS (positive effect)	“Shamanism is a complex set of practices, often involving ceremonies, initiations, mythologies, and music. Thus, like other forms of complex culture, it can disappear following demographic fragmentation, isolation, and bottlenecks (Henrich 2004; Shennan 2001).” (Singh, 2017)	Consumer Group Size<br>Max Camp Size<br>Total Population Size
**11**	Resource stress → RS (positive effect)	“The shaman may also be asked to relieve community suffering from such things as a natural disaster, poor harvest, social discord, hunger, or illness. Individuals may seek out the shaman when suffering from sickness or misfortune. In either situation, the shaman’s function is to communicate with the ancestors or spirits who may be responsible for the perils being suffered." (Rossano, 2007)<br><br>“According to this theory, and the writings of many other researchers (Malinowski 1948), magical practices result from individuals trying to control important, uncontrollable events. As individuals are better able to deal with this uncertainty – or if they confront less of it – they will depend less on shamans. Although a world completely devoid of uncertain outcomes seems implausible,	Mean Environmental Productivity

		Hart and Pilling (1960) offered this hypothesis to explain the absence of shamanism among the Tiwi. Citing the adequate rainfall, the consistent food supply, a dearth of dangerous animals, the rarity of tropical diseases, climatic docility, and the absence of antagonistic neighbors, the ethnographers concluded, "[The Tiwi] never invented magic to control their environment because their physical environment was on the whole a satisfactory and not a hostile universe" (p. 88)." (Singh, 2017)	
**12**	Natural disasters → RS (positive effect)	"The shaman may also be asked to relieve community suffering from such things as a natural disaster, poor harvest, social discord, hunger, or illness. Individuals may seek out the shaman when suffering from sickness or misfortune. In either situation, the shaman's function is to communicate with the ancestors or spirits who may be responsible for the perils being suffered." (Rossano, 2007)<br><br>"According to this theory, and the writings of many other researchers (Malinowski 1948), magical practices result from individuals trying to control important, uncontrollable events. As individuals are better able to deal with this uncertainty – or if they confront less of it – they will depend less on shamans. Although a world completely devoid of uncertain outcomes seems implausible, Hart and Pilling (1960) offered this hypothesis to explain the absence of shamanism among the Tiwi. Citing the adequate rainfall, the consistent food supply, a dearth of dangerous animals, the rarity of tropical diseases, climatic docility, and the absence of antagonistic neighbors, the ethnographers concluded, "[The Tiwi]	Environmental Predictability

		never invented magic to control their environment because their physical environment was on the whole a satisfactory and not a hostile universe” (p. 88).” (Singh, 2017)	
**13**	Resource unpredictability → RS<br>(positive effect)	“The shaman may also be asked to relieve community suffering from such things as a natural disaster, poor harvest, social discord, hunger, or illness. Individuals may seek out the shaman when suffering from sickness or misfortune. In either situation, the shaman’s function is to communicate with the ancestors or spirits who may be responsible for the perils being suffered." (Rossano, 2007)<br><br>“According to this theory, and the writings of many other researchers (Malinowski 1948), magical practices result from individuals trying to control important, uncontrollable events. As individuals are better able to deal with this uncertainty – or if they confront less of it – they will depend less on shamans. Although a world completely devoid of uncertain outcomes seems implausible, Hart and Pilling (1960) offered this hypothesis to explain the absence of shamanism among the Tiwi. Citing the adequate rainfall, the consistent food supply, a dearth of dangerous animals, the rarity of tropical diseases, climatic docility, and the absence of antagonistic neighbors, the ethnographers concluded, “[The Tiwi] never invented magic to control their environment because their physical environment was on the whole a satisfactory and not a hostile universe” (p. 88).” (Singh, 2017)	Environmental Predictability

**14**	Illness prevalence → RS (positive effect)	“The shaman may also be asked to relieve community suffering from such things as a natural disaster, poor harvest, social discord, hunger, or illness. Individuals may seek out the shaman when suffering from sickness or misfortune. In either situation, the shaman’s function is to communicate with the ancestors or spirits who may be responsible for the perils being suffered." (Rossano, 2007)<br><br>“According to this theory, and the writings of many other researchers (Malinowski 1948), magical practices result from individuals trying to control important, uncontrollable events. As individuals are better able to deal with this uncertainty – or if they confront less of it – they will depend less on shamans. Although a world completely devoid of uncertain outcomes seems implausible, Hart and Pilling (1960) offered this hypothesis to explain the absence of shamanism among the Tiwi. Citing the adequate rainfall, the consistent food supply, a dearth of dangerous animals, the rarity of tropical diseases, climatic docility, and the absence of antagonistic neighbors, the ethnographers concluded, “[The Tiwi] never invented magic to control their environment because their physical environment was on the whole a satisfactory and not a hostile universe” (p. 88).” (Singh, 2017)	Pathogen Load
**15**	Seasonal resource variation → RS (positive effect)	“According to this theory, and the writings of many other researchers (Malinowski 1948), magical practices result from individuals trying to control important, uncontrollable events. As	Environmental Variation<br><br>Environmental Predictability

individuals are better able to deal with this uncertainty – or if they confront less of it – they will depend less on shamans. Although a world completely devoid of uncertain outcomes seems implausible, Hart and Pilling (1960) offered this hypothesis to explain the absence of shamanism among the Tiwi. Citing the adequate rainfall, the consistent food supply, a dearth of dangerous animals, the rarity of tropical diseases, climatic docility, and the absence of antagonistic neighbors, the ethnographers concluded, “[The Tiwi] never invented magic to control their environment because their physical environment was on the whole a satisfactory and not a hostile universe” (p. 88).” (Singh, 2017)

“The shaman may also be asked to relieve community suffering from such things as a natural disaster, poor harvest, social discord, hunger, or illness. Individuals may seek out the shaman when suffering from sickness or misfortune. In either situation, the shaman’s function is to communicate with the ancestors or spirits who may be responsible for the perils being suffered." (Rossano, 2007)

Table S2. Bivariate relationships between professional religious specialists and other variables. These results summarize the results of a generalized least squares models, without controlling for the non-independence of societies.

**Variable**	**Estimate**	**Std. Error**	*z*	**n**	*p*
Soc. Class	2.42*	1.07	2.27	72	0.023
Slavery	1.74	1.09	1.60	72	0.110
Internal Conflict	0.13	0.56	0.24	70	0.809
Intergroup Conflict	0.90	0.53	1.71	71	0.088
Warfare	1.33*	0.54	2.49	72	0.013
Food Storage	2.75***	0.69	3.98	72	<0.001
Leadership	2.56***	0.69	3.73	72	<0.001
N. Annual Moves	0.03	0.28	0.09	58	0.924
Dist. Annual Moves	0.12	0.29	0.40	53	0.692
Consumer Group Size	0.66	0.34	1.95	68	0.051
Max Camp Group size	0.65	0.37	1.78	54	0.075
Total Pop. Size	0.47	0.26	1.79	72	0.073
Pathogens	-1.02**	0.31	-3.33	72	0.001
Mean Environmental Productivity	-0.50*	0.25	-1.98	72	0.048
Environmental Predictability	0.83**	0.29	2.85	72	0.004
Environmental Variation	0.32	0.25	1.25	72	0.211

* Indicates a *p* value of less than 0.05, ** indicates a *p* value of less than 0.01, and *** indicates a *p* value of less than 0.001

Table S3. Summary of the 30 best fitting model structures. Twenty seven models are within 2 CICc of the lowest CICc.

**Model ID**	**k**	**q**	**C**	**p**	**CICc**	**Delta CICc**	**l**	**w**
m22224	5	16	5.82	0.830	48.49	0.00	1.00	0.015
m100947	5	16	6.05	0.811	48.72	0.23	0.89	0.013
m22256	5	16	6.19	0.799	48.86	0.37	0.83	0.012
m100884	5	16	6.21	0.797	48.88	0.39	0.82	0.012
m21216	6	15	9.66	0.646	48.89	0.40	0.82	0.012
m21280	6	15	10.03	0.614	49.26	0.77	0.68	0.010
m21222	5	16	6.78	0.746	49.44	0.96	0.62	0.009
m18528	7	14	13.57	0.482	49.49	1.00	0.61	0.009
m47904	6	15	10.37	0.584	49.60	1.11	0.57	0.008
m21286	5	16	6.95	0.730	49.62	1.13	0.57	0.008
m67565	4	17	3.47	0.901	49.71	1.23	0.54	0.008
m47968	6	15	10.49	0.573	49.72	1.23	0.54	0.008
m22230	4	17	3.51	0.899	49.75	1.26	0.53	0.008
m18592	7	14	13.94	0.454	49.86	1.37	0.50	0.007
m67534	4	17	3.64	0.888	49.88	1.39	0.50	0.007
m66591	5	16	7.31	0.696	49.97	1.49	0.48	0.007
m21224	5	16	7.34	0.693	50.01	1.52	0.47	0.007
m66528	5	16	7.47	0.680	50.14	1.65	0.44	0.006
m100892	4	17	3.90	0.866	50.14	1.65	0.44	0.006
m100955	4	17	3.90	0.866	50.14	1.65	0.44	0.006
m21456	6	15	11.00	0.529	50.23	1.74	0.42	0.006
m22262	4	17	4.04	0.854	50.28	1.79	0.41	0.006
m99508	6	15	11.11	0.519	50.34	1.85	0.40	0.006
m63904	6	15	11.22	0.510	50.45	1.96	0.37	0.005
m21233	4	17	4.23	0.836	50.47	1.98	0.37	0.005
m66597	4	17	4.23	0.836	50.47	1.99	0.37	0.005
m18536	6	15	11.25	0.507	50.48	2.00	0.37	0.005
m99444	6	15	11.27	0.506	50.51	2.02	0.36	0.005
m21288	5	16	7.87	0.641	50.54	2.05	0.36	0.005
m18534	6	15	11.36	0.498	50.59	2.10	0.35	0.005

Table S4. Summary of the average causal path model summarizing the results of the best four fitting models. Following von Hardenberg & Gonzalez-Voyer (Hardenberg & Gonzalez-Voyer, 2013), we report conditional averaging in which the path coefficients are only averaged across models in which the path appears.

			95% CI	
**Path**	**Coefficient**	**S.E.**	**lower**	**upper**
Env. Rich. -> Soc. Lead.	0.332	0.124	0.089	0.575
Env. Pred. -> Soc. Lead.	0.32	0.184	-0.04	0.68
Path. Load. -> Soc. Lead.	-0.462	0.229	-0.911	-0.013
Prof. Rel. Spec. -> Soc. Lead.	0.105	0.073	-0.037	0.248
Food Stor. -> Soc. Lead.	0.46	0.126	0.213	0.707
Env. Pred. -> Env. Rich.	-0.895	0.088	-1.067	-0.722
Env. Rich. -> Env. Pred.	-0.683	0.067	-0.815	-0.552
Env. Rich. -> Path. Load.	-0.327	0.101	-0.526	-0.128
Env. Pred. -> Path. Load.	-0.98	0.116	-1.208	-0.753
Soc. Lead. -> Prof. Rel. Spec.	0.061	0.146	-0.226	0.347
Env. Rich. -> Prof. Rel. Spec.	-0.126	0.123	-0.366	0.115
Food Stor. -> Prof. Rel. Spec.	0.549	0.145	0.265	0.833
Soc. Lead. -> Food Stor.	0.307	0.09	0.131	0.483
Env. Rich. -> Food Stor.	-0.086	0.07	-0.224	0.051
Path. Load. -> Food Stor.	-0.738	0.144	-1.02	-0.456

Supplementary References

Atkinson, J. M. (1992). Shamanisms today. *Annual Review of Anthropology*, *21*(1), 307–330.

Binford, Lewis, R. (2001). *Constructing frames of reference: An analytical method for archaeological theory building using ethnographic and environmental data sets*. University of California Press.

Botero, C. A., Gardner, B., Kirby, K. R., Bulbulia, J., Gavin, M. C., & Gray, R. D. (2014). The ecology of religous beliefs. *Proceedings of the National Academy of Sciences*, *111*(47), 16784–16789.

Colwell, R. K. (1974). Predictability, constancy, and contingency of periodic phenomena. *Ecology*, *55*(5), 1148–1153.

Dow, M., & Eff, E. (2008). Global, regional, and local network autocorrelation in the standard cross-cultural sample. *Cross-Cultural Research*, *42*(2), 148–171.

Fincher, C. L., & Thornhill, R. (2008). Assortative sociality , limited dispersal , infectious disease and the genesis of the global pattern of religion diversity. *Proceedings of the Royal Society B: Biological Sciences*, *275*(1651), 2587–2594. https://doi.org/10.1098/rspb.2008.0688

Fincher, C. L., Thornhill, R., Murray, D. R., & Schaller, M. (2008). Pathogen prevalence predicts human cross-cultural variability in individualism/collectivism. *Proceedings of the Royal Society B: Biological Sciences*, *275*(1640), 1279–1285.

Fritz, S. A., & Purvis, A. (2010). Selectivity in mammalian extinction risk and threat types: A new measure of phylogenetic signal strength in binary traits. *Conservation Biology*, *24*(4), 1042–1051.

Fukuyama, F. (2011). *The origins of political order: From prehuman times to the French Revolution*. Farrar, Straus and Giroux.

Hammarström, H., Forkel, R., Haspelmath, M., & Bank, S. (2020). *Glottolog 4.3*. Max Planck Institute for the Science of Human History. https://doi.org/10.5281/zenodo.4061162

Hardenberg, A. von, & Gonzalez-Voyer, A. (2013). Disentangling evolutionary cause-effect relationships with phylogenetic confirmatory path analysis. *Evolution: International Journal of Organic Evolution*, *67*(2), 378–387.

Hayden, B., & Villeneuve, S. (2010). Who benefits from complexity? A view from Futuna. In *Pathways to power* (pp. 95–145). Springer.

Ho, L. S. T., Ane, C., Lachlan, R., Tarpinian, K., Feldman, R., Yu, Q., van der Bijl, W., Maspons, J., Vos, R., & Ho, M. L. S. T. (2016). Package 'phylolm.' *See Http://Cran. r-Project. Org/Web/Packages/Phylolm/Index. Html (Accessed February 2018)*.

Jordan, F. M. (2013). Comparative phylogenetic methods and the study of pattern and process in kinship. In P. McConvell, I. Keen, & R. Hendery (Eds.), *Kinship systems: Change and reconstruction* (pp. 43–58). University of Utah Press.

Kirby, K. R., Gray, R. D., Greenhill, S. J., Jordan, F. M., Gomes-Ng, S., Bibiko, H. J., Blasi, D. E., Botero, C. A., Bowern, C., Ember, C. R., Leehr, D., Low, B. S., McCarter, J., Divale, W., & Gavin, M. C. (2016). D-PLACE: A global database of cultural, linguistic and environmental diversity. *PLoS ONE*, *11*(7).

Mace, R., & Pagel, M. (1994). The comparative method in anthropology. *Current Anthropology*, *35*(5), 549–564. https://doi.org/10.1086/204317

Orme, D., Freckleton, R., Thomas, G., Fritz, S. A., Isaac, N., & Pea, W. (2013). *Caper: Comparative Analyses of Phylogenetics and Evolution in R*.

Peoples, H. C., Duda, P., & Marlowe, F. W. (2016). Hunter-Gatherers and the Origins of Religion. *Human Nature*, *26*, 261–282. https://doi.org/10.1007/s12110-016-9260-0

Petrullo, V. (1939). *The Yaruros of the Capanaparo River, Venezuela*. Washington: U.S. Govt Print. Off.

R Core Team. (2015). R: A Language and Environment for Statistical Computing. *R Foundation for Statistical Computing*.

Rossano, M. J. (2007). Supernaturalizing Social Life: Religion and the Evolution of Human Cooperation. *Human Nature*, *18*, 272–294.

Running, S. W., Nemani, R., Glassy, J. M., & Thornton, P. E. (1999). MODIS daily photosynthesis (PSN) and annual net primary production (NPP) product (MOD17) Algorithm Theoretical Basis Document. *University of Montana, SCF At-Launch Algorithm ATBD Documents (Available Online at: Www. Ntsg. Umt. Edu/Modis/ATBD/ATBD_MOD17_v21. Pdf)*.

Singh, M. (2017). The cultural evolution of shamanism. *Behavioral and Brain Sciences*, *41*, E66. https://doi.org/10.1017/S0140525X17001893

Testart, A., Forbis, R. G., Hayden, B., Ingold, T., Perlman, S. M., Pokotylo, D. L., Rowley-Conwy, P., & Stuart, D. E. (1982). The significance of food storage among hunter-gatherers: Residence patterns, population densities, and social inequalities. *Current Anthropology*, *23*(5), 523–537.

van der Bijl, W. (2018). phylopath: Easy phylogenetic path analysis in R. *PeerJ*, *6*, e4718.

Watts, J., Hamerslag, E. M., Sprules, C., & Dunbar, R. I. M. (2019). *Hunter-gatherer Religion Database (HGRD)*. Open Science Framework. http://dx.doi.org/10.17605/OSF.IO/XUJC6

Weber, M. (1958). The three types of legitimate rule. *Berkeley Publications in Society and Institutions*, *4*(1), 1–11.

Winkelman, M. (1986). Magico-Religious Practitioner Types and Socioeconomic Conditions. *Cross-Cultural Research*, *20*(17), 17–46.

Winkelman, M. (1989). A Cross-cultural study of Shamanistic Healers. *Journal of Psychoactive Drugs*, *21*(1), 17–24. https://doi.org/10.1080/02791072.1989.10472139

Winkelman, M. J. (1990). Shamans and other “magico-religious” healers: A cross-cultural study of their origins, nature, and social transformations. *Ethos*, *18*(3), 308–352.

Wright, R. (2010). *The Evolution of God: The origins of our beliefs*. Hachette.

Yu, V. L., & Edberg, S. C. (2005). Global Infectious Diseases and Epidemiology Network (GIDEON): A world wide Web-based program for diagnosis and informatics in infectious diseases. *Clinical Infectious Diseases*, *40*(1), 123–126.
